# Meiotic and pedigree segregation analyses in carriers of t(4;8)(p16;p23.1) differing in localization of breakpoint positions at 4p subband 4p16.3 and 4p16.1

**DOI:** 10.1007/s10815-015-0622-z

**Published:** 2015-12-04

**Authors:** Alina T. Midro, Marcella Zollino, Ewa Wiland, Barbara Panasiuk, Piotr S. Iwanowski, Marina Murdolo, Robert Śmigiel, Maria Sąsiadek, Jacek Pilch, Maciej Kurpisz

**Affiliations:** Department of Clinical Genetics, Medical University of Białystok, Waszyngtona 13, 15-089 Białystok, PO Box 22, Poland; Department of Medical Genetics, Università Cattolica Sacro Cuore, Rome, Italy; Department of Reproductive Biology and Stem Cells, Institute of Human Genetics, Polish Academy of Sciences, Strzeszyńska 32, 60-479 Poznań, Poland; Department of Genetics, Medical University of Wrocław, Wrocław, Poland; Department of Child Neurology, Medical University of Silesia, Katowice, Poland

**Keywords:** FISH mapping, Genetic counseling, Reciprocal chromosome translocation, Pedigree segregation analysis, Meiotic segregation pattern

## Abstract

**Purpose:**

The purpose of this study was to compare meiotic segregation in sperm cells from two carriers with t(4;8)(p16;p23.1) reciprocal chromosome translocations (RCTs), differing in localization of the breakpoint positions at the 4p subband—namely, 4p16.3 (carrier 1) and 4p16.1 (carrier 2)—and to compare data of the pedigree analyses performed by direct method.

**Methods:**

Three-color fluorescent in situ hybridization (FISH) on sperm cells and FISH mapping for the evaluation of the breakpoint positions, data from pedigrees, and direct segregation analysis of the pedigrees were performed.

**Results:**

Similar proportions of normal/balanced and unbalanced sperm cells were found in both carriers. The most common was an alternate type of segregation (about 52 % and about 48 %, respectively). Unbalanced adjacent I and adjacent II karyotypes were found in similar proportions about 15 %. The direct segregation analysis (following Stengel-Rutkowski) of the pedigree of carriers of t(4;8)(p16.1;p23.1) was performed and results were compared with the data of the pedigree segregation analysis obtained earlier through the indirect method. The probability of live-born progeny with unbalanced karyotype for carriers of t(4;8)(p16.1;p23.1) was moderately high at 18.8 %—comparable to the value obtained using the indirect method for the same carriership, which was 12 %. This was, however, markedly lower than the value of 41.2 % obtained through the pedigree segregation indirect analysis estimated for carriers of t(4;8)(p16.3;p23.1), perhaps due to the unique composition of genes present within the 4p16.1–4p 16.3 region.

**Conclusions:**

Revealed differences in pedigree segregation analysis did not correspond to the very similar profile of meiotic segregation patterns presented by carrier 1 and carrier 2. Most probably, such discordances may be due to differences in embryo survival rates arising from different genetic backgrounds.

## Introduction

Carriership of a reciprocal chromosome translocation (RCT) is one of the underlying conditions resulting in live-born offspring with chromosomal imbalances and may lead to unfavorable pregnancy outcomes such as miscarriages, stillbirths, and early newborn deaths. Clinical effects vary due to the different forms of the unbalanced gametes produced during the meiotic segregation of chromosomes involved in RCT. These different forms of unbalanced gametes can yield different survival rates of the unbalanced embryo, fetus, or child [1, 2]. Knowledge of the meiotic patterns of segregation of RCT carriers, as compared to empirical data on segregation from pedigree analysis, may be useful in illustrating the natural selection of children due to various chromosomal imbalances in different prenatal and postnatal periods of development. Such results may prove valuable for genetic counseling of RCT carrier families [2–4]. The probabilities of different unfavorable pregnancy outcomes for carriers of particular RCTs—as determined from pedigree analyses—depend strongly on the type, size, and genetic content of unbalanced chromosomal segments involved in RCT [1, 5–10]. The t(4;8)(p16;p23) translocations in humans are not unique, since they arise independently in unrelated individuals. This is due to the repetitive sequences (LTR) of the olfactory receptor (OR) gene clusters at 4p16.1 and 4p16.3 and also at 8p23.1. These repetitive sequences likely facilitate recurrent nonallelic homologous recombinations (NAHR) between both nonhomological chromosomes [11–14].

Our published preliminary data from the two pedigrees of t(4;8)(p16;p23) carriers with breakpoint positions at 4p16.3 and at 4p16.1 and 8p23.1 yielded a significantly higher probability of offspring with unbalanced karyotype in the case of t(4;8)(p16.3;p23.1) than in the case of t(4;8)(p16.1;p23.1), although both risks can be described as high [15]. In order to improve the preliminary assessment of the probability of the offspring of t(4;8)(16.1;p23.1) carriers having unbalanced karyotypes, in this study, new pedigree data were obtained for these t(4;8)(16.1;p23.1) carriers, allowing a direct segregation analysis with ascertainment correction. In order to better characterize each chromosome translocation, fluorescent in situ hybridization (FISH) mapping was used for the precise evaluation of the breakpoint positions. Since meiotic segregation was not investigated in the sperm of male carriers of t(4;8)(p16.3;p23.1) or of t(4;8)(p16.1;p23.1), we performed this analysis using the three-color FISH method. Next, the obtained results were compared to the data pedigree segregation analysis of carriers of the same RCT. Knowledge of the proportion of genetically unbalanced gametes produced by male carriers of t(4;8)(16.3;p23.1) and t(4;8)(p16.1;p23.1), separately, could be helpful in explaining the differences observed in the probability rates for children with unbalanced karyotypes at birth for either RCTs.

## Material and methods

### Studied group

#### Members of pedigree 1 (reported by Midro et al. [15])

Carrier 1 of t(4;8)(p16.3;p23.1)pat(pedigree 1, III:3) is a 34-year-old father of a boy with trisomy 4p16.3 → pter and monosomy 8p23.1 → pter. His pedigree, consisting of the cytogenetic and empirical data on 20 pregnancies in seven carriers’ families, has previously been reported. For FISH mapping, the breakpoint positions of blood lymphocytes of carrier 1’s brother’s daughter with trisomy 4p16.3 → pter and monosomy 8p23.1 → pter were used (child 1; previously reported as IV:3 in pedigree 1) [15].

#### Members of pedigree 2 (reported by Midro et al. [15])

Carrier 2 of a t(4;8)(p16.1;p23.1)dn (pedigree 2, II:3) is a 34-year-old father of a girl with Wolf–Hirschhorn syndrome (WHS) due to monosomy 4p16.1 → pter together with trisomy 8p23.1 → pter [der(4)t(4;8)(p16.1;p23.1)], previously reported among one family of members. For FISH mapping of the breakpoint positions, the blood lymphocytes of this girl (child 2) were used.

#### Members of pedigree 3

**Fig. 1:**
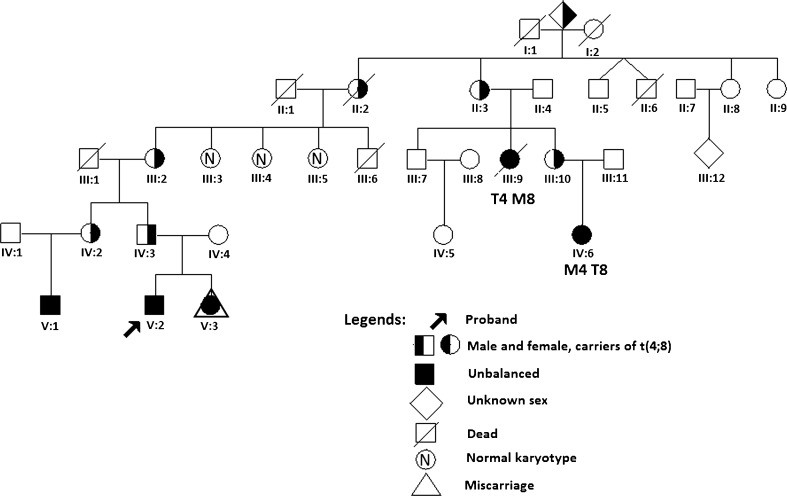
Investigated pedigree 3 of the family of t(4;8)(16.1;p23.1) carriers with legends

The pedigree of the family is shown in Fig. 1. The family was ascertained through karyotyping of a boy (V:2) with WHS phenotype in whom an unbalanced translocation der(4)t(4;8)(p16.1;p23.1)pat was found (child 3). Further family studies demonstrated der(8)t(4;8)(p16.1;p23.1) in a son (V:1) of his paternal sister (IV:2). His blood lymphocyte samples were used for the more precise identification of breakpoint positions with FISH mapping. In addition, the same imbalance was found in two paternal relatives (III:9; IV:6) and in one child at prenatal studies (V:3). Carriership of a balanced t(4;8)(p16.1;p23.1) was detected in a further five family members (IV:2; III:2,10; II:2–3, and probably have been in their progenitors ( I:1 or I:2) also.

### Fluorescence in situ hybridization (FISH mapping)

FISH mapping of breakpoint positions on chromosomes 4 and 8 involved in der(8)t(4;8)(p16.3;p23.1) and der(4)t(4;8)(p16.1;p23.1) was carried out on metaphase chromosomes using the cosmid and bacterial artificial chromosome (BAC) clones from the RPCI-11 library (Table 1). Molecular probes specific to the 4p and 8p regions were chosen from libraries available on the Web (UCSC, NCBI, Ensembl) and prepared as described elsewhere [14].

**Table 1:** Results of FISH mapping, using clones specific to 4p and 8p chosen from libraries available on the Web (UCSC, NCBI, Ensembl)

Probe	Distance from 4pter (Mb) or genome location (bp)^a^	Chromosome region	FISH signal on der(4)	FISH signal on der(8)	
			Child 2	Child 1	Child 3
4p					
pC847.351	0.15 Mb	4p16.3	−		
190b4	1.95 Mb		−		
108f12	2.2 Mb			+	
228a7	3.7 Mb			+	
RP11-323F5	chr4:4,543,858-4,546,634			−	
MSX1	chr4:4,861,392-4,865,660	4p16.2	−		
RP11-524D9	5.9 Mb		−		
RP11-301J10	chr4:8,469,579-8,636,558	4p16.1	−		+
RP11-423D16	chr4:8,520,479-8,687,458		−		+
RP11-751L19	chr4:9,721,722-9,886,942		+		−
RP11-731E20	chr4:13,323,555-13,495,132	4p15.33	+		
8p					
RP11-372K15	chr8:6,478,452-6,673,842	8p23.1	+	−	−
RP11-1195F20	chr8:7824866-7826066			+	+
RP11-403C10	chr8:9,633,438-9,815,348		−	+	+

^a^In accordance with the Human Feb 2009 (GRCh37/hg19) Assembly

### Semen collection and preparation

Semen samples of carriers 1 and 2 with normozoospermia were collected by masturbation and spontaneously liquefied at room temperature. Sperm cells were washed in BWW medium [16] and fixed with methanol/acetic acid (3:1) as previously described [3, 4]. Sperm nuclear decondensation was performed in 10 mM dithiothreitol (DTT, Sigma) in 0.1 M Tris–HCl (pH 8.0) for 5–10 min at 43 °C. The semen slides were rinsed in 2× sodium chloride/sodium citrate (2× SCC; pH 7.0) and air-dried following an ethanol dehydration series.

### DNA probes and FISH procedure

**Fig. 2:**
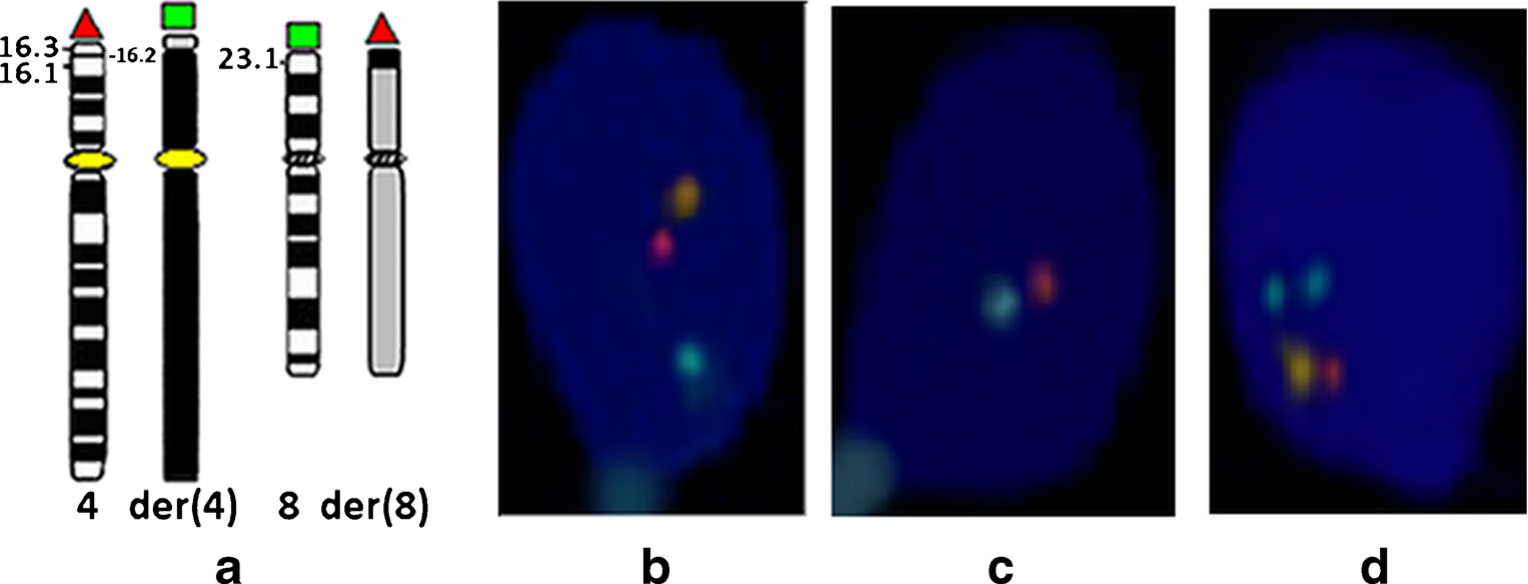
Schematic representation of breakpoint positions (*left*) with marked position of tri-color FISH probes: *orange*—centromere of chromosome 4, *red*—4p, *green*—8p (**a**) and examples of FISH signals corresponding to segregation sperm products of the t(4;8)(16.1;p23.1) carrier: **b** sperm normal/balanced genotype after alternate 2:2 segregation; **c** sperm unbalanced genotype 23,-4,+der8 after adjacent 2 (2:2) segregation; **d** sperm unbalanced genotype 24,-4,+der4,+der8 after 3:1 segregation

Three-color FISH was performed with directly labeled probes (Cytocell, Cambridge, UK) to analyze the meiotic segregation pattern, as has been previously described [3, 4]. A combination of two centromeric probes of chromosome 4 (4c Green plus 4c Red for receiving the yellow color) and two telomeric probes (4p Red and 8p Green) was used. The position of the FISH probes is shown in Fig. 2a. The FISH analyses were performed in line with the manufacturer’s instructions. The hybridization mixture (2.5 μl of each probe, 10 μl of hybridization buffer) was applied to the slide, covered with a cover slip, and sealed with rubber cement. The combined denaturation of probes and spermatozoa was performed for 2.5 min at 75 °C on a slide warmer. Hybridization was carried out overnight in a dark humidified chamber at 37 °C. The slides were then washed for 2 min in a solution of 0.4× SCC at 72 °C and then for 30 s in a solution of 2× SSC/0.05 % Tween 20. Counterstaining was performed with 4′,6-diamidino-2-phenylindole (DAPI). Hybridization signals were observed using an Olympus Bx41 microscope fitted with a triple-bandpass filter for DAPI/FITC/Texas Red. Subsequent image acquisition was performed using a digital color camera and ISIS software (in situ imaging system) (MetaSystems, v. 5.0, Altussheim, Germany). The meiotic segregation pattern was analyzed in 4000 sperm nuclei by determining the presence or absence of the FISH signals corresponding to chromosomes 4, 8, der(4), and der(8). The efficiency of hybridization was about 97 %. Strict scoring criteria were applied: two signals of the same color were counted as such only if they were separated by at least one domain. It should be noted that, in the case of sperm from translocation carriers, the efficiency of hybridization was evaluated indirectly: parallel to the sperm of the t(4;8)(p16.1;p23.1) carrier, control FISH were also performed (under the same experimental conditions and with the same probes) on sperm from a volunteer with normal somatic karyotype. In the donor’s case, the efficiency of hybridization was calculated by counting only the number of hybridized spermatozoa with three signals (yellow + red + green meaning normal sperm karyotype) in the randomly scored 500 spermatozoa.

### Probability rates estimation

Based on segregation analysis of cumulative data from pedigree 2 [15] and pedigree 3, the probability rate for children having unbalanced karyotype at birth and for children having normal phenotype at birth were calculated according to the direct method with ascertainment correction of Stengel-Rutkowski et al. [6] and Daniel [17]. The ascertainment correction consists of elimination of probands (or index sibships) and carriers with the proband in direct line of descent.

## Results

### FISH mapping of breakpoint positions

#### der(8)t(4;8)(p16.3;p23.1) (child 1 from pedigree 1 published by Midro et al. [15])

**Fig. 3:**
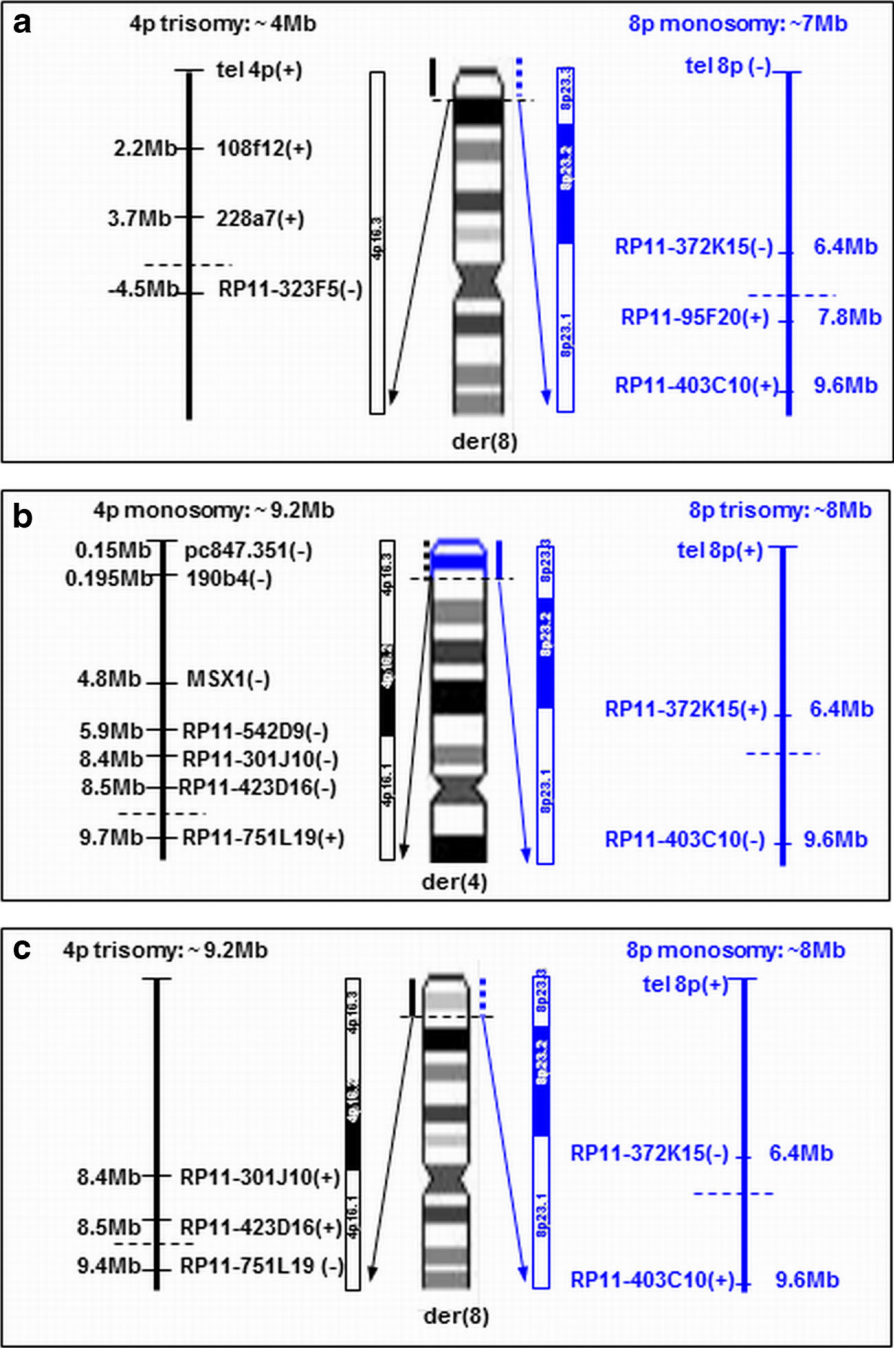
**a**–**c** FISH mapping of breakpoint positions. Explanations are presented in Table 1

The breakpoint position at 4p16.3 was mapped between two BAC clones: RP11-323F5 (mapping on 4p at about 4.5 Mb from telomere) absent on der(8) and clone 228a7 (mapping on 4p at about 3.7 Mb from telomere) present on der(8). The breakpoint position on 8p occurred at 8p23.1 between BAC clones RP11-372K15 (mapping on 8p at about 6.6 Mb from the telomere) absent on der(8) and RP11-1195F20 (mapping on 8p at about 9.7 Mb from telomere) present on der(8) (Fig. 3a, Table 1). The unbalanced karyotype in the form of partial trisomy 4p16.3 → pter (about 4 Mb in size) and partial monosomy 8p23.1 → pter (about 7 Mb in size) was in this way diagnosed in child 1.

#### der(4)t(4;8)(p16.1;p23.1) (child 2 from pedigree 2 published by Midro et al. [15])

The breakpoint position at 4p16.1 was mapped between two BAC clones RP11-423D16 (9 Mb from telomere) absent on der(4) and RP11-751L19 (9.4 Mb from telomere) present on der(4). The breakpoint at 8p23.1 was mapped between the BACs RP11-372K15 (mapping on 8p at about 6.6 Mb from telomere) and RP11-403C10 (mapping on 8p at about 9.7 Mb from telomere) (Fig. 3b, Table 1). The unbalanced karyotype in the form of partial monosomy 4p16.1 → pter (about 9.2 Mb in size) and partial trisomy 8p23.1 → pter (about 8 Mb in size) was thus diagnosed in child 2.

#### der(4)t(4;8)(p16.1;p23.1) (child 3 from pedigree 3 in Fig. 1)

The breakpoint position at 4p16.1 was mapped between two BAC clones: RP11-423D16 (9 Mb from telomere) present on der(8) and RP11-751L19 (9.4 Mb from telomere) absent on der(8). On the other hand, the breakpoint position at 8p23.1 was mapped between RP11-403C10 (mapping on 8p at about 9.7 Mb from telomere) present on der(8) and RP11-372K15 clone (mapping on 8p at about 6.6 Mb from the telomere) absent on der(8) (Fig. 3c, Table 1). A double chromosomal imbalance, consisting of partial trisomy 4p16.1 → pter (about 9.2 Mb in size) and partial monosomy 8p23.1 → pter (about 8 Mb in size), was diagnosed in child 3.

### Meiotic segregation pattern analysis by FISH

The results of the analysis of sperm cell meiotic segregation in the t(4;8)(p16.3;p23.1) and t(4;8)(p16.1;p23.1) carriers obtained from three-color FISH are shown in Table 2 and examples of FISH on spermatozoa with different genotypes are shown in Fig. 2. The most common was an alternate type of segregation: normal and balanced karyotypes were found in 51.9 % (carrier 1) and 52.4 % (carrier 2) of sperm nuclei. Adjacent I and adjacent II karyotypes were found in similar proportions in the sperm cells of both carriers (carrier 1, 15.5 and 14.3 %; carrier 2, 15.1 and 13.6 %). The total incidence of tertiary segregants was 7.8 % in carrier 1 and 8.2 % in carrier 2, but the incidence of 3:1 interchangeable segregants was almost half that in both carriers, 4.0 and 4.6 %, respectively.

**Table 2:** Frequencies (%) of different types of segregation in sperm from carrier 1 of t(4;8)(p16.3;p23.1) and carrier 2 of (4;8)(p16.3;p23.1)

Type of segregation	Carrier 1: t(4;8)(p16.3;p23.1)			Carrier 2: t(4;8)(p16.1;p23.1)		
	Sperm genotype	Percent	Total %	Sperm genotype	Percent	Total %
2:2						
Alternate	23 or 23,-4,-8,+der4,+der8	51.9	51.9	23 or 23,-4, −8,+der4,+der8	52.4	52.4
Adjacent I	23,-4+der4	7.8	15.5	23,-4+der4	8	15.1
	23,-8,+der8	7.7		23,-8,+der8	7.1	
Adjacent II	23,-8,+der4	6.6	14.3	23,-8,+der4	6.4	13.6
	23,-4,+der8	7.8		23,-4,+der8	7.2	
3:1						
Tertiary	24,+der4	1.5	3.8	24,+der4	1.1	2.7
	22,-4,-8,+der8	2.3		22,-4,-8,+der8	1.6	
	24,+der8	1.7	4	24,+der8	2.2	5.5
	22,-4,-8,+der4	2.3		22,-4,-8,+der4	3.3	
Interchange	24,-8,+der4,+der8	0.7	2.1	24,-8,+der4,+der8	0.9	2.5
	22,-4	1.4		22,-4	1.6	
	24,-4,+der4,+der8	0.6	1.9	24,-4,+der4,+der8	0.9	2.1
	22,-8	1.3		22,-8	1.2	
Unexplained			6.4			6.1

### Pedigree segregation analysis

The results of the direct pedigree segregation analysis (with ascertainment correction) of the families of the t(4;8)(p16.1;p23.1) carriers are presented in Table 3.

**Table 3:** The probability rates for live-born child with chromosomal imbalance—Rate^M^—and for the birth of child with normal phenotype—Rate^N^—of maternal (MAT), paternal (PAT), and unknown parental (MAT/PAT) origin for carriers of t(4;8)(p16.1;p23.1)

No.	Carrier	Parental origin of RCT	Live-born child with chr. imbl^M^		Child with normal phenotype at birth^N^		Pregnancies		Rate^M^	Rate^N^
			T	C	T	C	T	C		
Pedigree 2 [15]										
1.	II:3	PAT	1	0	1	1	2	1	0/1	1/1
Pedigree 3										
2.	IV:3	PAT	1	0	–	–	1	0	0/0	–/0
3.	IV:2	MAT	1	1	–	–	1	1	1/1	–/1
4.	III:2	MAT	–	–	2	1	2	1	–/1	1/1
5.	III:10	MAT	1	1	–	–	1	1	1/1	–/1
6.	II:2	MAT	–	–	5	4	5	4	–/4	4/4
7.	II:3	MAT	1	1	2	2	3	3	1/3	2/3
8.	I:1, I:2	MAT or PAT	–	–	6	5	6	5	–/5	5/5
Total			5	3	16	13	21	16	3/16	13/16
									18.8 ± 9.7 %	81.3 ± 9.7 %

*T* total number live-born children with chromosomal imbalance, children with normal phenotype at birth, and total number of pregnancies; *C* number of live-born children with chromosomal imbalance, children with normal phenotype at birth, and number of pregnancies after ascertainment correction

From a total of 21 pregnancies of 8 carriers of t(4;8)(p16.1;p23.1) in cumulative data from the two pedigrees (pedigree 2 and pedigree 3), we found 5 children with unbalanced karyotype (three with monosomy 4p/trisomy 8p: one girl II:3 in pedigree 2, boy V:2 and girl IV:6 in pedigree 3; three children (one boy and two girls) with trisomy 4p/monosomy 8 including one in prenatal diagnosis: V:1, III:9, V:3 in pedigree 3) and 16 children with normal phenotype at birth. Five members were omitted after ascertainment of the correction, namely: the female proband of pedigree 2 (III:3), the male proband of pedigree 3 (V:2), his father (IV:3), grandmother (III:2), and great-grandmother (II:2) (see Fig. 1). Finally, we found that the probability rate for birth of a malformed child with unbalanced karyotype for MAT/PAT carriers was 3/16 (18.8 ± 9.7 %). The probability rate for a child with normal phenotype at birth was 13/16 (81.3 ± 9.7 %) (Table 3). All available information on unfavorable pregnancy outcomes—such as miscarriages, stillbirths, and early newborn deaths—was requested.

## Discussion

Previous segregation analysis of pedigree data demonstrated generally high probabilities of the birth of a child with an unbalanced t(4;8)(p16;p23) karyotype [15]. Thus, our previous study of families of t(4;8)(p16.3;p23.1) carriers showed a value of 41.2 ± 11.9 % (7/17, pedigree 1, direct method analysis) [15], whereas the present study of families of t(4;8)(p16.1;p23.1) carriers indicated the probability to be somewhat lower, at about 18.8 ± 9.7 % (3/16, pedigree 3, direct method of analysis). This latter result corresponds well with the results of an indirect pedigree analysis of t(4;8)(p16.1;p23.1) carriers, which yielded a risk of about 12 %, as earlier described [15], and is also in line with the about 15 % risk calculated by Tranebjerg et al. [18]. These high probabilities rate for birth of child with chromosome imbalancy are not surprising. In general the relative high survival rates for progeny with imbalance of subterminal region of other involved chromosomes are observed in families of RCT carriers [2, 7, 19]. However, direct values of the probability rate for having children with unbalanced karyotype vary depending on the translocation (i.e., 18 %, compared to 42 %). The precise identification of breakpoint positions is therefore necessary for individual RCTs, as has been presented in this study.

To date, published data on meiotic segregation patterns is available for about 200 carriers of different RCTs and shows that the development of progeny with the onset of the genetic imbalance at meiosis can be variable [20–24]. The profile of meiotic segregation in both carriers of t(4;8)(p16.3 or p16.1;p23.1) revealed almost all types of segregants (neither 4:0 segregation nor recombinants were found) (Table 2). Interestingly, a similar proportion of normal/balanced to unbalanced sperm cells (about 52 % to about 48 %) was found in both carriers. Slightly more meiotic adjacent I segregants than adjacent II segregants (15.5 versus 14.3 % in carrier 1 and 15.1 versus 13.6 % in carrier 2) were encountered in our study (Table 2). Adjacent I segregation may lead to the development of viable progeny of t(4;8)(p16;p23.1) carriers with unbalanced karyotypes (monosomy 4p with trisomy 8p or trisomy 4p with monosomy 8p). These two possible forms of imbalance were observed in live-born progeny in the examined families and have also been described by others [6, 11, 13, 14, 18, 25–27]. The frequency of adjacent I segregants (about 15 %) corresponds well with results of the pedigree analysis of t(4;8)(p16.1;p23.1) carriers, which yield a risk of unbalanced live-born progeny of about 18–12 %, but it is markedly lower than the high risk (about 40 %) calculated for carrier of t(4;8)(p16.3;p23.1) [15].

The observation that the risk of live-born children having an unbalanced karyotype (trisomy or monosomy 4p16 with monosomy or trisomy 8p23.1) after a 2:2 disjunction and adjacent I segregation was approximately twice as great in families of t(4;8)(p16.3;p23.1) carriers than in families of t(4;8)(p16.1;p23.1) carriers is very intriguing. Pedigree analyses of 46 families with RCT involving 4p by Stengel-Rutkowski et al. [6] showed a high risk (20.5 ± 4.6 %) of unbalanced offspring (trisomy or monosomy 4p16) for RCT carriers of shorter segments and lower risks of about 4.5 ± 2.5 % for RCT carriers at risk of longer segment imbalances. This supports the results calculated for RCT carriers with other chromosomes, where the probability of unbalanced offspring generally increases with the decreasing length of the segments involved in RCT [7–10, 19].

Altogether, there is a clear discordance between the varying risk estimates calculated from pedigree segregation analyses compared to profiles of similar meiotic segregation in the two carriers t(4;8)(p16.3;p23.1) and t(4;8)(p16.1;p23.1) examined. We can speculate that this discordance may be due to the differences in embryonic survival rates caused by disparate genetic background. This could be due to different genetic content between 4p16.1 and 4p16.3 and encompassing about 100 genes. One possibility may indicate *MSX1 locus* (4p16.2) responsible for diminished embryonic survival due to impaired cardiovascular development [28].

Other factors involved can be based on information collected from family members. Unfortunately, any informations on unfavorable pregnancy outcomes like of miscarriages, stillbirths, early newborn deaths were done. Therefore the putative meiotic malsegregation responsible for limited survival in utero has not been confirmed. We cannot rule out the possibility that the high risk of unfavorable pregnancy outcomes observed in families of carriers of t(4;8)(p16.3;p23.1) observed by Tranebjaerg et al. [18] could be due to a limited prenatal survival rate on account of another form or forms of unbalanced progeny resulting, for example, from adjacent 2 or 3:1 segregations, as have been observed by us in meiotic studies of both carriers.

Similar profiles of meiotic segregation in gametes of both t(4;8) carriers with different breakpoint positions (at 4p16.3 or 4p16.1) could indicate that a small change in the breakpoint position at 4p16 (by one subband) does not impact the pattern of meiotic malsegregation. It should be emphasized that segregation pattern studies using three-color FISH (the technique most often applied) have their limitations. Among 16 different fluorescent signals (arising from spermatozoa karyotypes following the first meiotic division), only 14 can be differentiated under a microscope using this method. Sperm cells developing at 4:0 segregation (with no FISH signals) thus cannot be discriminated from sperm artifacts (as a rule, we have to consider hybridization efficiency below 100 %; in our studies, this was 97 %). A 4:0 segregation, however, is very rare. Moreover, spermatozoa that develop as a result of alternate segregation with normal chromosomes or balanced chromosomes have the same fluorescent phenotype but it could be assumed that these develop in a 1:1 proportion. It should also be emphasized that, in cases of alternate, adjacent I, and 3:1 segregations, three-color FISH does not differentiate recombination products in chromosome interstitial segments [20]. Awareness of certain limitations in meiotic segregation analysis by FISH (including hybridization efficiency) allows to estimate the error of the analysis a few percent. Despite such problems, we are confident that the final score illustrates well the meiotic segregation pattern in sperm cells of a particular RCT carrier.

To the best of our knowledge, the frequencies of particular types of meiotic segregation in the sperm of t(4;8)(p16.3;p23.1) and t(4;8)(p16.1;p23.1) carriers have not been described by others, so our results cannot be independently verified.

## Conclusions

In general, published data presenting both pedigree analysis and meiotic segregation patterns of male RCT carriers involving other chromosomes are scarce [2–4, 29]. However, in case of the lack of pedigree data, we may at least imply probabilities for birth child with balanced karyotype from the gamete meiotic segregation figures without risk of overestimating. However, for the probability rate estimates of a defined type of unbalanced progeny, it is essential to acquire information on its viability, in order to provide more specific prognostic data for genetic counseling [3].
